# A Table of Sounds

**Published:** 1881-03

**Authors:** 


A Table of Sounds by which the various heart troubles may be correctly and easily diagnosed, has been prepared by Prof. F. Peyre Porcher, M. D., of Charleston, S. C. We referred to the formulated arrangement of these sounds in a previous issue of this journal, and now publish it for the benefit of our readers. As it has been prepared by one of the most thoroughly cultured and intelligent gentlemen in the profession, it is entitled to the fullest confidence and respect of all. He says, "our table is very easily constructed, and being based upon eminently natural and scientific foundations, namely: the physical laws of the heart's structure, functions, and actions, it must serve as a ready method, enabling us, or any one else, even the most uninstructed, to make a diagnosis of all the uncomplicated organic diseases of the valves at the orifices of all the chambers of the heart. As it is necessarily true and correct, and though it may seem very simple, it requires no little thought to apply it to any case before us; or that we should at the time of applying it, understand *why* it is correct.

The formula and the order of words to be re-called are:—

*Stenosis.*

*Insufficiency.*

*Insufficiency.*

*Stenosis.*

For example:—

At the base. { A deranged 1st sound indicates *Stenosis* of the aorta, or pulmonary artery valves. A deranged 2d sound indicates *insufficiency* of the aorta, or pulmonary artery valves.

At the apex. { A deranged 1st sound indicates *Insufficiency* of the bicuspid, or of the tricuspid valves. A deranged 2d sound indicates *Stenosis* of the bicuspid or of the tricuspid valves.

All we have to do is to memorize these words in their order, as a formula, to elucidate at the bedside, the valvular disease of the heart. Observe what sounds are deranged at the base, then at the apex, and pronounce accordingly. Of course, the known relative positions of the four valves must guide us in deciding which of the two valves at the base, or at the apex, the abnormal murmur proceeds from, so as to distinguish between the valvular arrangements of the right and left heart. Dr. Alexander Harvey's formulæ are added also, and are as follows, viz:

Bruit: If *systolic* and loudest at *Base*—Aortic obstruction.

At *Apex*—Mitral Insufficiency.

Bruit: If *diastolic* and loudest at *Base*—Aortic Insufficiency. At Apex—Mitral obstruction.

This may be represented on a card carried in the pocket, and on the reverse side of card, thus:

Pulse: If regular,<br>

Full or strong, } Aortic Disease.<br>

Jerking, resistent.

Pulse: If irregular,<br>

Intermittent, unequal, } Mitral Disease.<br>

Soft, small, weak.

With the foregoing knowledge at command, an inexperienced practitioner would be enabled to reach important conclusions in heart troubles.

